# Books

**Published:** 1882-02

**Authors:** 


### Books.

---

A Treatise on Food and Dietetics Physiologically and Therapeutically Considered. By F. W. Pavy, M.D., F.R.S. Second Edition. New York: William Wood & Co. 1881. Wood's Library of Standard Medical Authors.

The author says in the preface to the first edition of this work, "From the fact that the subject of food is one of deep concern, both to the healthy and the sick—that the information, which has been obtained during the last few years, has completely revolutionized some of the cardinal scientific notions formerly entertained—and that no modern systematic treatise of the kind here presented exists in the English language—I have been encouraged to think that the task I have undertaken may not be superfluous."

The contents of this interesting book are systematically arranged, and the several divisions of the subject are fully and pleasingly treated. The first chapter is devoted to remarks on the dynamic relations of foods; then follow chapters on the origination of food, alimentary principles, alimentary substances, the preservation of food, principles of dietetics, practical dietetics, therapeutical dietetics, and hospital dietaries.

Referring to the origin of food the author reminds us, "That all organic matter has its primary source in the vegetable kingdom, from which kingdom, it follows, all our food must directly or indirectly be derived. The vegetable feeder goes directly for its food to the vegetable kingdom. The animal feeder is equally dependent upon the products of the vegetable kingdom for its pabulum. But it obtains it at second hand, so to speak, or in an indirect manner, its food consisting of the flesh of animals which have themselves been nourished upon vegetable products." Carbon, hydrogen, oxygen and nitrogen form by far the chief constituents of organic compounds, but sulphur and phosphorus are also present, to a small extent, bound up with the other elements in certain organic principles; and in vegetable products we find, not only the organic, but likewise the inorganic matter we require.

In discussing condensed milk as an article of diet this important and practical suggestion is quoted in a foot-note, "Whilst it is admitted that infants take it readily on account of its sweetness, grow plump, and appear to thrive remarkably well upon it, it is alleged that the appearance, which depends simply upon an accumulation of fat, is delusive, and that they in reality possess so little power that they become prostrated by diarrhœa and other affections, and rapidly sink in a manner that is not observed under other modes of feeding." The merits of white and brown bread are compared, and the conclusion is stated that the loss of the phosphates by excluding the outer portions of the grain would be a serious thing if bread was our only article of diet, but in an ordinary mixed diet the loss is abundantly compensated by the use of other forms of food.

Beverages receive a due share of attention. Alcohol, in the various forms in common use, is declared to be beneficial, when properly taken, in promoting digestion, improving nutrition and checking waste. Tea and coffee form favorite and useful beverages. The properties they possess fully justify the estimation in which they are held. They produce invigorating and restorative effects on the system, without being followed by any depression. Coffee is heavier and more oppressive to the stomach than tea.

In the chapter on therapeutic dietetics we find the following paragraph: "Liebig's extractum carnis is largely sold, and, from the prestige afforded by its inventor's name, has obtained a world-wide notoriety. Its true position is scarcely that of an article of nutrition, and this is now beginning to be generally recognized. The article, indeed, is free from albumen, gelatine and fat, and may be said to comprise the salines of the meat, with various extractive principles, a considerable portion of which, doubtless, consists of products in a state of retrograde metamorphosis, and of no use as nutritive agents. If not truly of alimentary value, the preparation, nevertheless, appears to possess stimulant and restorative properties which render it useful in exhausted states of the system."

The book contains a large amount of entertaining, useful and practical information, conveniently grouped and properly classified. The time spent in a careful examination of its teachings will undoubtedly be profitably employed.

---

Artificial Anæsthesia and Anæsthetics. By Henry M. Lyman, A.M., M.D. New York: William Wood & Co. 1881. Wood's Library of Standard Medical Authors.

This book represents the successful attempt of the author to produce a complete history of the subject in hand, distilled, he says, from the writings of the most trustworthy and capable observers in the domain of artificial anæsthesia. The first portion of the work relates to the history of anæsthesia. Dr. Crawford W. Long's connection with the early use of ether, for anæsthetic purposes, is faithfully detailed, and the date of its use by him, for the first time in a surgical operation—March, 1842—is correctly stated. However, according to the statement of the writer, the administration of ether by inhalation, for the prevention of pain, was first successfully accomplished by William T. G. Morton, who, in January, 1842, administered the ether to a patient from a folded towel, for a dentist in Rochester, N. Y., and a tooth was extracted without pain.—Page 6. This declaration is at variance with, and is irreconcilable with the conclusions arrived at by Dr. J. Marion Sims after a patient, laborious and painstaking investigation of the whole subject of priority in the discovery of anæsthesia, and we shall not apologize for quoting, in connection with this claim, these conclusions.* Dr Sims says:

"We know, 1st. That since 1800, the inhalation of nitrous oxide gas produced a peculiar intoxication, and even allayed headache and other minor pains.

*Virginia Medical Monthly, May, 1877.

2d. That Sir Humphrey Davy proposed it as an anæsthetic in surgical operations.

3d. That for more than fifty years the inhalation of sulphuric ether has been practiced by the students of our New England colleges as an excitant, and that its exhilarating properties are similar to those of nitrous oxide gas.

4th. That the inhalation of sulphuric ether, as an excitant, was common in some parts of Georgia forty-five years ago, though not practiced in the colleges.

5th. That Wilhite was the first man to produce profound anæsthesia, which was done accidentally with sulphuric ether, in 1839.

6th. That Long was the first man to intentionally produce anæsthesia for surgical operations, and that this was done with sulphuric ether in 1842.

7th. That Long did not by accident hit upon it, but that he reasoned it out in a philosophical and logical manner.

8th. That Wells, without any knowledge of Long's labors, demonstrated in the same philosophic way, the great principle of anæsthesia by the use of nitrous oxide gas—1844.

9th. That Morton intended to follow Wells in using the gas as an anæsthetic in dentistry, and for this purpose asked Wells to show him how to make the gas—1846.

10th. Wells referred Morton to Jackson for this purpose, as Jackson was known to be a scientific man and an able chemist.

11th. That Morton called on Jackson for information on the subject, and that Jackson told Morton to use sulphuric ether instead of nitrous oxide gas, as it was known to possess the same properties, was as safe and easier to get.

12th. That Morton, acting upon Jackson's off-hand suggestions, used the ether successfully in the extraction of teeth—1846.

13th. That Warren and Haygood and Bigelow performed important surgical operations in the Massachusetts General Hospital—October 1846—on patients etherized by Morton, and that this introduced and popularized the practice throughout the world."

There really seems to be some inconsistency in the record made by Professor Lyman concerning Morton's first use of ether, for, after making the assertion above cited, namely, that Morton produced anæsthesia from ether in January, 1842, on the same page, we read: "In the year 1846, an enterprising dentist in the city of Boston, William T. G. Morton by name, was occupying his brain with the idea of painless dentistry. The memory of his experience in the year 1839, and his conversations with apothecaries and chemists—notably with Dr. Charles Jackson, an expert chemist, led him to experiment with ether by inhalation. On the evening of Sept. 30, 1846, he put himself to sleep with ether, and when he recovered consciousness, he found that he had been insensible for eight minutes. This was the result for which he had hoped, and the first patient who entered his office, a boy named Eben Frost, was persuaded to undertake a repetition of the experiment," etc. etc.

Now, if he had achieved such signal success in 1842, wherefore the necessity of obtaining hints and suggestions from chemists and others concerning ether and anæsthesia in 1846? and why was any doubt entertained respecting the effect of the drug, when he used it on himself before using it on the boy, Eben Frost?

The last paragraph accords accurately with the evidence adduced by Dr. Sims, but the first statement is seemingly at varience with both the following paragraph and with Dr. Sims' conclusions.

A list of forty-seven anæsthetic agents is given, and each one is more or less fully described. Of these the most important are ether and chloroform, and, comparing these two, the writer says, "the first is still preferred in the country where its utility was originally made known—the last is more highly valued in England and Europe; but the palm of safety must always remain with ether—the simplest, the surest, the safest of all the potent anæsthetic agents that are known among men."

The medico-legal relations of anæsthesia form one of the most interesting chapters in the book. Some of the evidence here collated sustains the popular belief that chloroform may be administered during sleep in such a way as to facilitate the commission of crime, but it further shows that this result is not likely to happen in the ordinary, unskilled use of the anæsthetic.

The volume of Professor Lyman will occupy an important place in the useful series of works, issued annually, by the enterprising publishers.

---

A SYSTEM OF SURGERY. Theoretical and practical, in treatises by various authors. Edited by T. Holmes, A.M., Cantab. First American from second English edition. Revised by Jno. H. Packard, A.M., M.D., assisted by a large corps of American surgeons. In three volumes. Henry C. Lea's Son & Co.: Philadelphia.

It has been but a short time since the first volume of this work was reviewed in the REGISTER. The second volume follows closely upon the first. The third will complete the whole work. The present volume is divided into four parts, devoted to diseases of the organs of special sense, of the circulatory system, of the digestive tract, the genito-urinary organs, and the surgical diseases of women. The various parts and chapters are written by some of the most eminent English surgeons, and in this issue are revised by such American surgeons as Harlan, Burnett, Cohen, McBurney, Stimson, Busey, White, Truman, Sims, Packard, Keyes and Skene.

The whole work is elaborate and exhaustive. Each subject, so far as our examination has gone, is thoroughly treated. The latest and most approved views are given, from both the English and American stand-point. We must confess we turn with more pleasure and confidence to the American comments than to the original English text. This, though, may arise from life-time training, and from greater familiarity with these writers. While our space will not allow us to make such comments as might be appropriately made, generally favorable, but now and then the reverse, we still may say that the consideration of aneurism is remarkably well done, both by Mr. Callender, the English author, and Dr. Stimson, the American editor. The chapter on hernia, by Jno. Birkett, Esq., and edited by Dr. Jno. H. Packard, of Philadelphia, we must also say is extremely well presented. The chapters on diseases of the genito-urinary organs, by Sir Henry Thompson, and revised by Dr. Edward L. Keyes, one would expect to be thoroughly satisfactory—and so they are. We can scarcely conceive of a better selection of author and reviser for this especial work. Our impression is that the chapter on surgical diseases of women is scarcely up to the general average of the work; it certainly does not confer upon the reader that sense of completeness derived from the part just commented on. Here, too, it seems the deficiency, if such there be, rests rather with J. Hutchinson, Esq., than with the reviser, A. J. C. Skene, M.D.

In conclusion, this may be pronounced a great work. Imperfections, should one search for them, may be found in any book; but he will probably find as few here as in any other. A physician supplied with this work has a library of surgical knowledge on which he may confidently rely for reference.

The paper and printing (in double column) is well done; the binding is good; the illustrations are copious and satisfactory; and the general make-up pleasing.

